# The Spectral Diversity of Resting-State Fluctuations in the Human Brain

**DOI:** 10.1371/journal.pone.0093375

**Published:** 2014-04-11

**Authors:** Klaudius Kalcher, Roland N. Boubela, Wolfgang Huf, Lucie Bartova, Claudia Kronnerwetter, Birgit Derntl, Lukas Pezawas, Peter Filzmoser, Christian Nasel, Ewald Moser

**Affiliations:** 1 Center for Medical Physics and Biomedical Engineering, Medical University of Vienna, Vienna, Austria; 2 MR Centre of Excellence, Medical University of Vienna, Vienna, Austria; 3 Department of Statistics and Probability Theory, Vienna University of Technology, Vienna, Austria; 4 Department of Psychiatry and Psychotherapy, Medical University of Vienna, Vienna, Austria; 5 Department of Radiodiagnostics and Nuclear Medicine, Medical University of Vienna, Vienna, Austria; 6 Department of Psychiatry, Psychotherapy and Psychosomatics, RWTH Aachen University, Aachen, Germany; 7 Department of Radiology, Tulln Hospital, Tulln, Austria; 8 Brain Behaviour Laboratory, Department of Psychiatry, University of Pennsylvania Medical Center, Philadelphia, Pennsylvania, United States of America; Max Planck Institute for Human Cognitive and Brain Sciences, Germany

## Abstract

In order to assess whole-brain resting-state fluctuations at a wide range of frequencies, resting-state fMRI data of 20 healthy subjects were acquired using a multiband EPI sequence with a low TR (354 ms) and compared to 20 resting-state datasets from standard, high-TR (1800 ms) EPI scans. The spatial distribution of fluctuations in various frequency ranges are analyzed along with the spectra of the time-series in voxels from different regions of interest. Functional connectivity specific to different frequency ranges (<0.1 Hz; 0.1–0.25 Hz; 0.25–0.75 Hz; 0.75–1.4 Hz) was computed for both the low-TR and (for the two lower-frequency ranges) the high-TR datasets using bandpass filters. In the low-TR data, cortical regions exhibited highest contribution of low-frequency fluctuations and the most marked low-frequency peak in the spectrum, while the time courses in subcortical grey matter regions as well as the insula were strongly contaminated by high-frequency signals. White matter and CSF regions had highest contribution of high-frequency fluctuations and a mostly flat power spectrum. In the high-TR data, the basic patterns of the low-TR data can be recognized, but the high-frequency proportions of the signal fluctuations are folded into the low frequency range, thus obfuscating the low-frequency dynamics. Regions with higher proportion of high-frequency oscillations in the low-TR data showed flatter power spectra in the high-TR data due to aliasing of the high-frequency signal components, leading to loss of specificity in the signal from these regions in high-TR data. Functional connectivity analyses showed that there are correlations between resting-state signal fluctuations of distant brain regions even at high frequencies, which can be measured using low-TR fMRI. On the other hand, in the high-TR data, loss of specificity of measured fluctuations leads to lower sensitivity in detecting functional connectivity. This underlines the advantages of low-TR EPI sequences for resting-state and potentially also task-related fMRI experiments.

## Introduction

From the first identification of intrinsic fluctuations in fMRI signal in the resting brain, these have been consistently described as low-frequency fluctuations in the range of 0.01 to 0.1 Hz [1]–[3]. This definition is based primarily on two reasons, one of them technical and the other being related to the physiological model used to describe blood oxygenation level dependent (BOLD) fluctuations in the brain [4]. The technical reason is the upper limit of the sampled frequencies in the sequences usually employed, typically allowing only for the analysis of the behaviour of BOLD fluctuations below about 0.25 Hz (for a TR of 2 s). The physiological reason is the delay and temporal smoothing introduced by the hemodynamic response, which neuronal activation is subject to before being detectable by fMRI. This delay is in the range of 3 to 10 seconds [5], [6], and it follows that BOLD signal changes due to neuronal events filtered by the hemodynamic response function are to be found in a frequency range below about 0.15 Hz.

From a technical point of view, faster scanning has been possible for a long time only under rather restricted circumstances, for example when using a limited brain coverage like a single slice. Using such methods, it was possible to investigate in detail the temporal dynamics of the BOLD response to specific events [7]–[9], but not all applications were amenable to investigation under restricted fields of view. In particular, the field of resting-state fMRI is focused on the connectivity of spatially distant regions in coherent resting-state networks, which may only be analyzed with whole-brain coverage – this has long been beyond the reach of fast measuring techniques. Until recently, most fMRI measurements were performed with TRs of between 1 and 3 s, which means that the highest frequencies that can be critically sampled range between 1.67 and 0.5 Hz. Even then, there were indications that resting-state network fluctuations could be identified in higher frequencies than 0.1 Hz [10], [11].

It is only with the advent of more advanced fMRI imaging techniques that a more comprehensive investigation of the high-frequency dynamics of BOLD fluctuations in the human brain can be undertaken. Multiband imaging [12], [13] has allowed EPI imaging to be performed with higher spatial and/or temporal resolution, and both lead to quite different applications. The increase in temporal resolution has made it feasible to acquire images of the whole brain at reasonable spatial resolutions with subsecond repetition times. Though there exist other techniques yielding even higher temporal resolutions [14], [15], this increase comes at the cost of lower effective image resolution. In contrast, Feinberg et al. [12] found that functional connectivity in resting-state networks was improved when using multiband EPI image acquisition with TRs under 1 s compared to non-accelerated EPI with a TR of 2.5 s, indicating that the loss in effective image resolution is outweighed by the gain in temporal resolution, at least in the context of connectivity measures. Furthermore, the identification of resting-state functional modes using multiband imaging for higher temporal resolution has established the multiband imaging technique as a robust choice for the analysis of resting-state data [16].

Faster fMRI techniques have allowed to perform analyses on resting-state data including higher frequencies, leading to generally promising and sometimes unexpected results. For example, Zuo et al. [17] have investigated the regional homogeneity (ReHo) of resting-state fluctuations and found that its reliability is increased with fast sampling rates. Liao et al. [18] have calculated graph based metrics on resting-state data with a higher temporal resolution and not only found low-frequency network characteristics consistent with previous low-TR findings, but also revealed several default-mode regions to be network hubs at higher frequencies (0.2–0.3 Hz). Finally, Boubela et al. [19] identified resting-state networks using ICA on high-frequency fluctuations (0.25–1.4 Hz) and revealed that even though their peak power is at low frequencies, about 50% of fluctuation amplitude of resting-state networks occur at frequencies higher than 0.25 Hz. These results suggest that high-frequency BOLD fluctuations during rest are not only consisting of nuisance factors, but contain information that can lead to a deeper understanding of resting-state fluctuations themselves.

A thorough investigation of these high-frequency dynamics, however, has not yet been performed. Baria et al. [20] have explored the temporal dynamics of BOLD fluctuations in high-TR resting-state data and found that the prevalence of different frequencies varied across brain regions, corroborating earlier results that the power spectra of resting-state oscillations varied across the brain [21]. In these studies, it was found that the relative power at higher frequencies was higher in subcortical regions as well as in the insula, while low-frequency fluctuations were most prevalent in regions in the prefrontal, parietal and occipital cortex. These analyses, however, have been limited to frequencies lower than 0.2 Hz, while oscillations of higher frequencies have not been mapped. In the present study, we set out to close this gap and provide a groundwork for further investigations of the high-frequency BOLD dynamics in the resting human brain.

## Results

**Figure 1:**
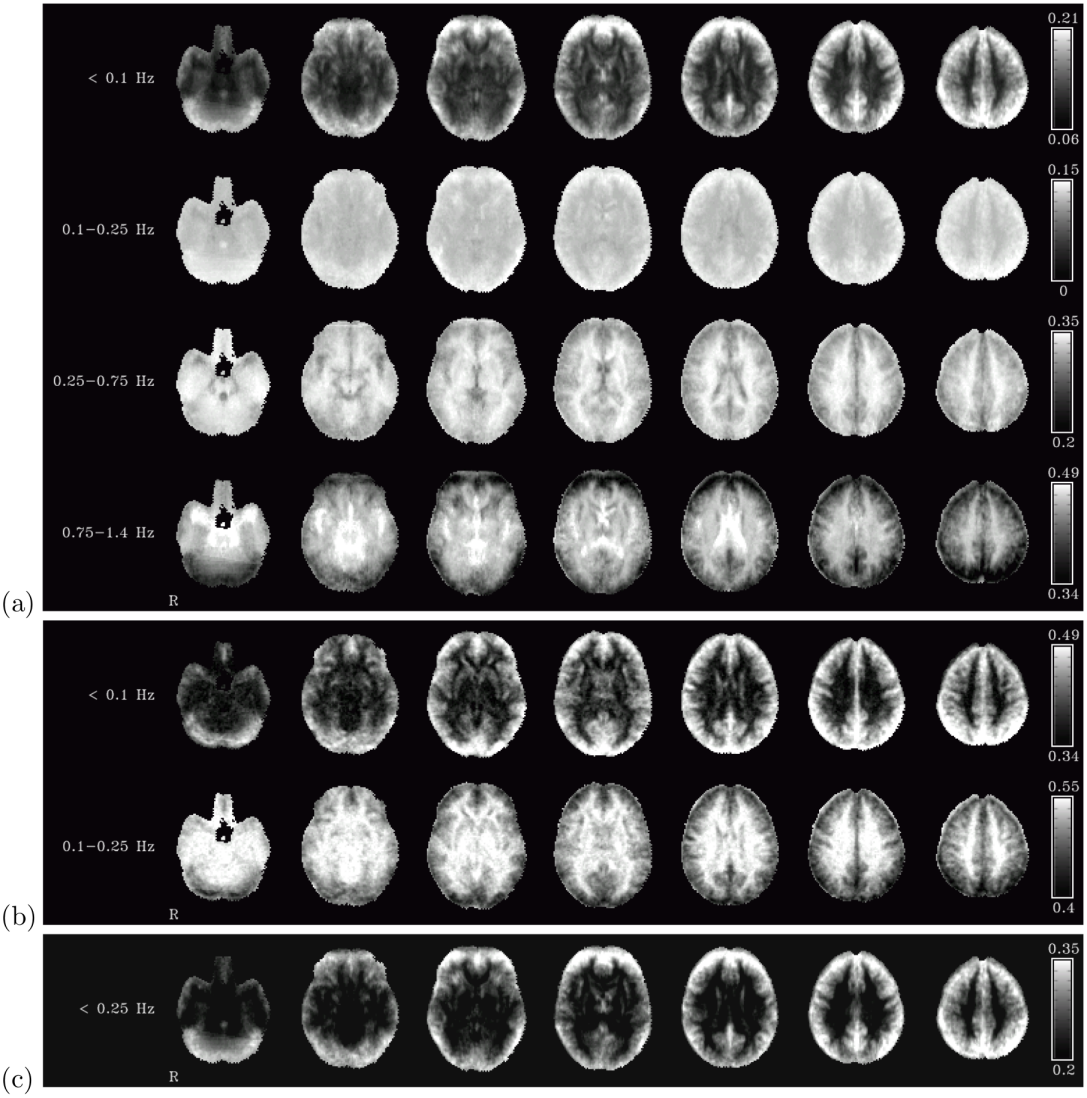
Fractional amplitudes of fluctuations. Fractional amplitudes of fluctuations in four different frequency bands in the low-TR dataset (a), in two different frequency bands in the high-TR dataset (b), and in the two lower frequency bands combined in the low-TR dataset (c). All color bars are set to the same window width (0.15).

The main differences in the spatial distribution of the fractional amplitudes of different frequency bands are between cerebro-spinal fluid (CSF), white matter and grey matter (see figure 1 (a)). In the frequency band below 0.1 Hz, grey matter areas had highest relative fluctuation amplitude (with values exceeding 20% in some voxels), whereas the relative fluctuation amplitude of oscillations of this range in both white matter and CSF were minimal (about 5–10% in most voxels). Average relative fluctuation amplitudes were 15% in grey matter and 9–10% in white matter and CSF (see table 1). In the next frequency range under consideration (i.e., the band between 0.1 and 0.25 Hz corresponding to frequencies measured with high-TR measurements but usually eliminated in resting-state analyses by the band-pass filtering employed in many studies) there was still a moderately high proportion of the fluctuation amplitude in the grey matter (around 15%), and about the same amount in white matter and CSF as in the lowest frequency range (around 10%).

**Table 1:** Mean spectral power proportions.

Low-TR	Grey Matter	White Matter	Ventricles
<0.1 Hz	**0.15**	0.09	0.10
0.1–0.25 Hz	**0.13**	0.11	0.11
0.25–0.75 Hz	0.32	**0.34**	0.30
0.75–1.4 Hz	0.41	0.46	**0.49**
**High-TR**	**Grey Matter**	**White Matter**	**Ventricles**
<0.1 Hz	**0.42**	0.35	0.36
0.1–0.25 Hz	0.50	**0.55**	**0.55**

Mean spectral power proportions in the indicated frequency bands within grey matter, white matter and ventricle masks in the low-TR (top) and high-TR (bottom) datasets. Voxelwise spectra were calculated and averaged across all voxels within the respective masks. Row-wise maxima are highlighted in the table. In the low-TR dataset, low-frequency fluctuations up to 0.25 Hz are most pronounced in grey matter, while medium and high frequency oscillations have highest amplitude in white matter and in the ventricles, respectively. In the high-TR dataset, fluctuations below 0.1 Hz are present predominantly in grey matter, and fluctuations between 0.1 and 0.25 Hz in white matter and ventricles.

The pattern reverses in the higher frequency ranges, though. In the frequency range between 0.25 and 0.75 Hz, the highest relative fluctuation amplitude was measured in the white matter (about 34% on average), while both grey matter and CSF showed lower amplitude (about 20–30%). Finally, in the highest band of frequencies, i.e. those above 0.75 Hz, highest relative amplitude was recorded in the CSF (49% on average); the oscillations in the white matter had slightly lower relative amplitude (about 46% on average). Even though oscillations in this frequency range were lower within grey matter as compared to all other regions, they still amounted to about 40% of total signal fluctuation on average. It should be noted, though, that high relative amplitude does not necessarily correspond to high absolute fluctuation amplitudes – in particular, the higher relative amplitude in the high frequency range in white matter is mostly due to the fact that there is very little absolute fluctuation amplitude in all frequency ranges, in contrast to the ventricles, where the high absolute fluctuation amplitude in the high frequency range is due to a distinct peak at these frequencies (see below).

Beside these general considerations, a number of more localized effects can be noted in figure 1 (a). Grey matter regions showed marked variability in the distribution of the frequencies of their signal oscillations. Subcortical regions showed lower fractional amplitude of low frequency fluctuations, and instead stronger fluctuations in the frequency ranges of 0.25–0.75 Hz (in particular for the basal ganglia) and above 0.75 Hz (in particular in the amygdala). The insula, and above all the posterior insula, exhibited a lower fluctuation amplitude than other cortical areas in the low frequency range, fluctuations of about the same amplitude than other cortical areas in the frequency range 0.25–0.75 Hz, and markedly higher amplitude in the frequency range above 0.75 Hz.

When comparing these percentual values with each other, one should bear in mind that the frequency bands chosen were not of equal width, and thus the wider bands (i.e., the two higher-frequency bands) are expected to yield higher relative fluctuation amplitudes than the lower frequency bands for this reason alone (even under the assumption of oscillations being uniformely distributed across all frequencies). While the maps in figure 1 (a) serve to visualize the spatial distribution of broad frequency patterns, the relative weight of the oscillations of each frequency band within the same region are better evaluated when viewing the spectra of the oscillations in particular regions.

**Figure 2:**
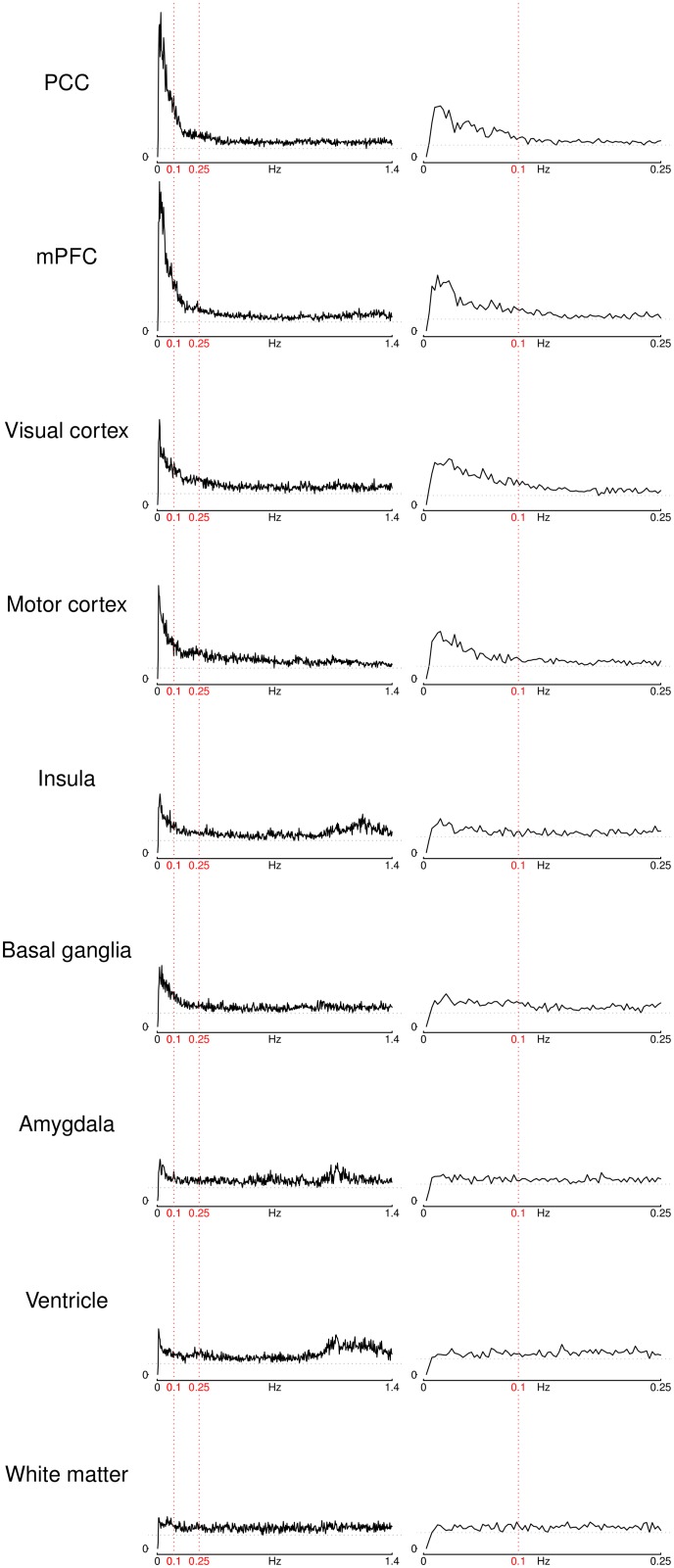
ROI power spectra. Power spectra of the center voxel time courses of selected ROIs for the low-TR dataset (left) and the high-TR dataset (right). Vertical red lines indicate 0.1 Hz and 0.25 Hz, dotted grey horizontal lines mark the minimum power in each plot. (mPFC stands for medial prefrontal cortex, PCC for posterior cingulate cortex).

Indeed, the patterns seen in figure 1 (a) are also reflected in the more detailed view of the power spectra of the time courses measured in 7 selected individual voxels shown in figure 2 (left column). Cortical grey matter voxels show heavily skewed power distributions, with most power at the lowest frequencies. The power then declines sharply towards higher frequencies, until only a small residual fluctuation amplitude remains (note that it still remains at a relatively constant low level, and never completely vanishes). It can also be seen that this decline is more pronounced in regions of the default mode network, i.e. the PCC and the mPFC, compared to other cortical regions like the visual cortex, the motor cortex or the insula, all three showing less steep a peak than the default-mode regions. In addition, one can see in the spectrum of the insula voxel that there is a second peak of fluctuation amplitude at about 1.2 Hz which, albeit lower than the first peak at the low frequencies, still accounts for a large proportion of the variation of the time course in this region. The amygdala voxel time course is similar to the insula in that it also has a peak at low frequencies and a second peak at about 1.2 Hz. The basal ganglia spectrum resembles the cortical spectra, but the low-frequency peak in this region is less pronounced than that of all cortical areas except for the insula.

The white matter power spectrum is distinct from all other spectra in that it has a relatively constant power across all frequency ranges except for a very slight increase in power in the lowest frequencies. The power spectrum in the ventricular voxel’s time course also has a slight peak at the lowest frequencies, but the frequency domain with the most pronounced oscillations in this voxel were at 1–1.4 Hz. It is worth noting at this point that the frequency range of this high-frequency peak is approximately the same for the ventricular voxel and for the insula and amygdala voxels.

These results can then be compared to the power spectra from the high-TR scans taken as a reference (see figure 2, right column). In regions where there is both a very pronounced peak at low frequencies and a constant, low baseline of fluctuation amplitude (i.e. in the mPFC, PCC, visual vortex and motor cortex), a peak at low frequencies and a subsequent decline of power towards higher frequencies can be observed, though the higher baseline amplitude in the visual and motor cortices means that the low frequency peak in this region is less pronounced compared to the default-mode regions. The spectrum of the basal ganglia lost the peak at low frequencies seen in the low-TR spectrum, and instead had a more even distribution across the frequency ranges sampled. In regions with a second peak of fluctuation amplitude above 1 Hz in the low-TR spectra, that is, the amygdala, insula as well as the ventricles, the high-TR spectra show an almost constant spectrum across the whole frequency range measured, likely due to the undersampling of the high-frequency oscillations which lead to a constant increase of fluctuation amplitude across all frequencies. Finally, the white matter voxel, which already had an almost constant spectrum in the low-TR data, also has a rather constant spectrum in the high-TR dataset, with even the slight increase in amplitude at lowest frequencies observed in the low-TR dataset vanishing here. To summarize the differences in the strengths of the low-frequency peaks, the ratio between the peak power at low frequencies and the minimum power at higher frequencies for all ROIs and for both TRs is given in table 2.

**Table 2:** ROI power ratios.

ROI	Ratio low-TR	Ratio high-TR
PCC	17.78	4.39
mPFC	17.14	4.82
Visual Cortex	7.75	5.04
Motor	8.83	3.79
Insula	4.86	2.14
Basal Ganglia	4.61	2.40
Amygdala	3.23	1.71
Ventricle	4.19	1.92
White Matter	2.37	1.67

Ratio between peak low frequency power and minimum power to the right of the peak for various ROIs. (PCC stands for posterior cingulate cortex, mPFC for medial prefrontal cortex).

In grey matter regions, the low-frequency peaks seen in the high-TR dataset can only be distinguished from the baseline up to approximately 0.1 Hz, while the low-frequency peaks in the low-TR dataset extend to higher frequencies, up to and in some cases even beyond 0.25 Hz (e.g., PCC, mPFC, visual and motor cortices). In other regions (insula, basal ganglia, amygdala), a low-frequency peak can only be distinguished in the low-TR dataset, while no clear peak is visible in the spectra of the high-TR data.

In terms of spatial distribution, the results of this change in the frequency spectra can be seen in figure 1 (b). In the low frequency band, the grey matter regions still exhibit highest fluctuation amplitude, but the gap between the relative amplitudes in these regions and those in white matter and CSF regions is narrower than in the low-TR data seen in figure 1 (a): while in the low-TR dataset the low-frequency range was more specific to grey matter, in the high-TR dataset a higher baseline of fluctuations in the low-frequency band can also be seen in the white matter and the ventricles. In the higher frequency range between 0.1 and 0.25 Hz, however, the pattern seen in the low-TR data is reversed for the high-TR dataset: here, relative fluctuation amplitude in white matter and CSF regions is higher than in grey matter regions. When analyzing the relative fluctuation amplitude of these two frequency bands combined in the low-TR dataset, an increased contrast between grey matter regions on one hand and white matter and ventricles on the other can be observed (see figure 1 (c)). This again might indicate that the high level of low-frequency fluctuations (below 0.25 Hz) in the high-TR dataset is an artifact of the slow acquisition and the folding of higher-frequency oscillations into the lower frequency range due to aliasing.

**Figure 3:**
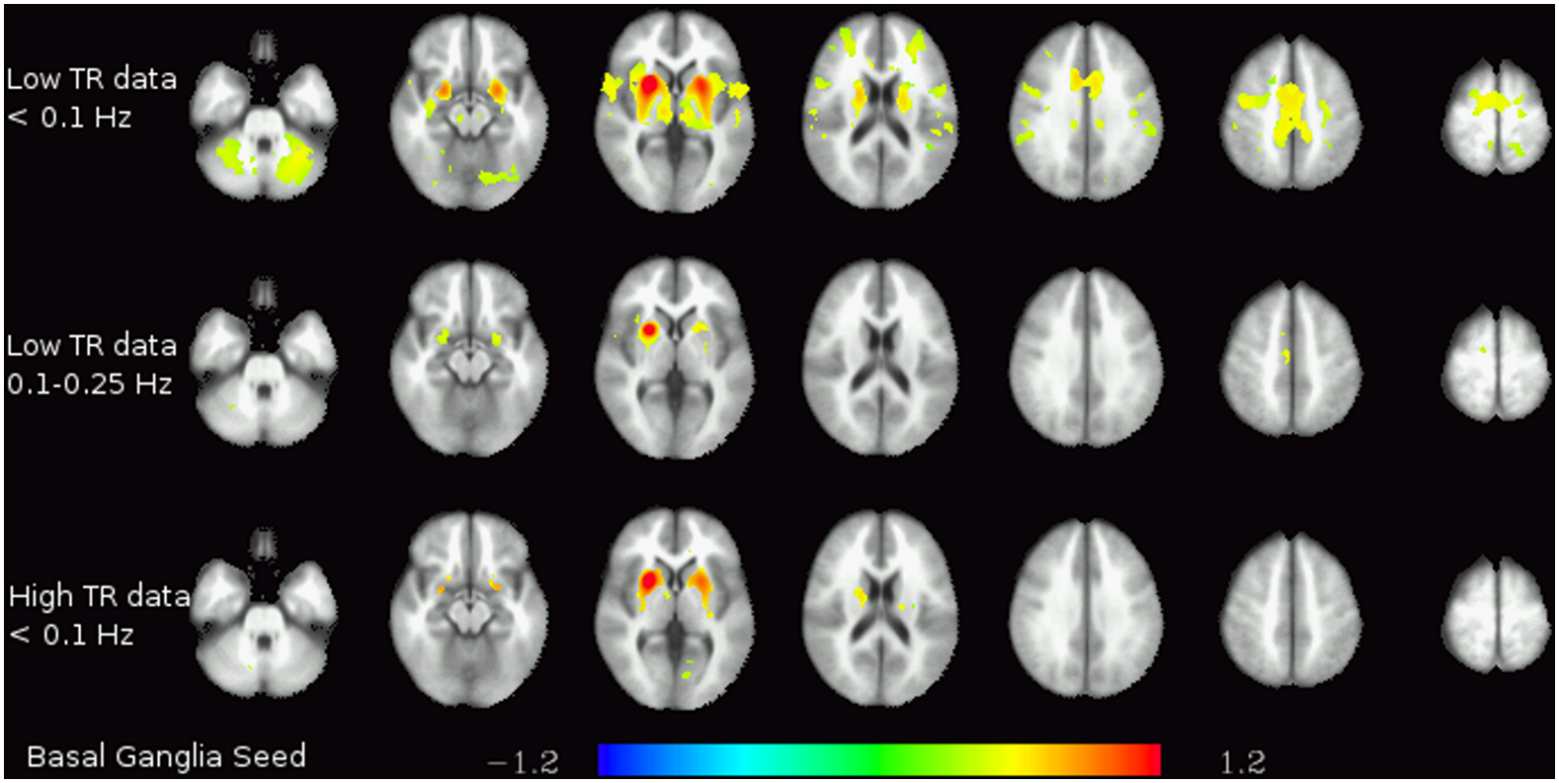
Basal ganglia functional connectivity. Functional connectivity of the basal ganglia seed in the lowest frequency band (<0.1 Hz) in the low-TR (top) and high-TR (bottom) datasets. The colors represent z-transformed correlation coefficients.

**Figure 4:**
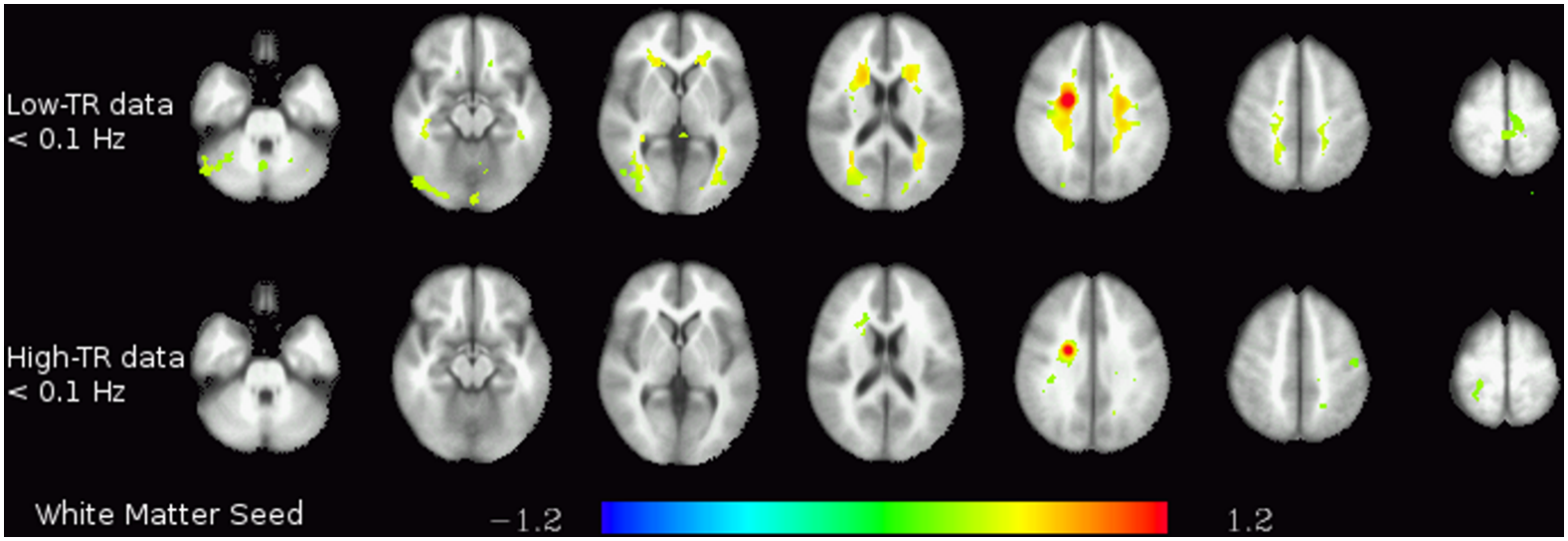
White matter functional connectivity. Functional connectivity of the white matter seed in the two lower frequency bands (<0.1 Hz and 0.1–0.25 Hz) in the low-TR (top) and high-TR (bottom) datasets. The colors represent z-transformed correlation coefficients.

Finally, the analyses on the functional connectivity in different frequency bands yielded some noteworthy differences between different frequency bands on one hand and between low-TR and high-TR data on the other. The first striking difference can be seen between the functional connectivity of the basal ganglia at frequencies <0.1 Hz in the datasets acquired at different TRs (see figure 3): in the low-TR data, significant connectivity not only to both left and right striatum, but also to the supplementary motor area (SMA), the mid-cingulate cortex, the thalamus and the cerebellum can be identified – the pattern corresponding to the motor loop known to be anatomically connected. In contrast, in the high-TR data, only the connectivity within the striatum itself can be observed. Analogously, there is significant connectivity in the low-TR data between the white matter seed and almost all other white matter regions in the brain in the frequency range <0.1 Hz (and no connectivity at higher frequencies), but no connectivity at all in the high-TR data (see figure 4).

**Figure 5:**
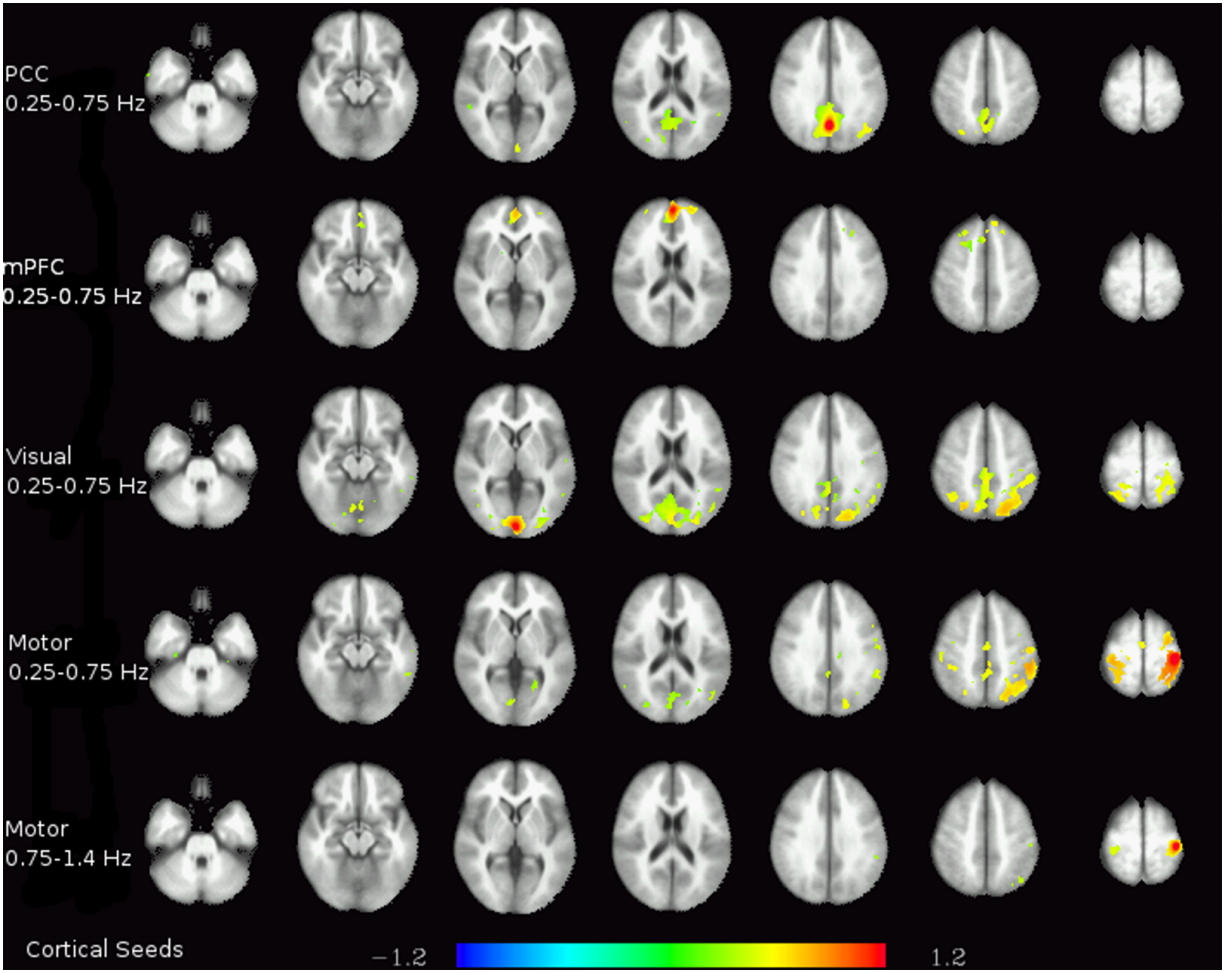
Functional connectivity of cortical regions in higher frequency bands. Each row shows the connectivity of the seed given on the left for the data band-passed to the frequency range noted there. The colors represent z-transformed correlation coefficients.

In the higher-frequency bands, interesting connectivity patterns can be seen in cortical grey matter regions (see figure 5), most importantly in the frequency band between 0.25 and 0.75 Hz. For the two default-mode seeds, significant connectivity can be observed to parts, but not all, of the DMN. The anterior seed in the mPFC showed significant connectivity to the prefrontal areas involved in the DMN, while the PCC seed showed significant connectivity to the precuneus and the lateral parietal cortices. Using the visual cortex seed, connectivity to (medial and lateral) parietal regions was found in the frequency band between 0.25 and 0.75 Hz. Among the cortical seeds used, the motor cortex seed was the only one to have significant connectivities even beyond 0.75 Hz, as the contralateral part of the motor cortex was still strongly correlated even in the highest frequency band.

## Discussion

This study shows that the dominant frequencies of spontaneous fluctuations vary greatly across different regions of the brain. Not only is there a large discrepancy between grey matter, white matter and CSF, but there is also considerable variability across different grey matter regions. One of the most important practical consequences of this result is that resting-state fluctuations in particular regions of the brain, most notably subcortical structures as well as the insula, cannot adequately be measured with a TR that is too long to critically sample frequency ranges with a relevant contribution to the spectrum in these regions [22].

The low-TR scans reveal that there are at least two frequency bands that contribute the bulk of the fluctuations in the brain, one being the low frequency range below 0.25 Hz, the other being a high-frequency range between 1 and 1.4 Hz. Broadly speaking, fluctuations in the lower frequency range are more pronounced in grey matter, and in particular in cortical regions of the brain, though some small amount of higher activity in this frequency range than in the higher frequency ranges can also be observed in the white matter as well as in the ventricles. The second, high-frequency range of large oscillations not only constitutes the largest source of fluctuations in the ventricles, but were also clearly visible in the spectra of the amygdala and the insula (see figure 2), potentially indicating physiological signal contamination. This is consistent with the arterial and venous vessel distribution in the brain [23].

Regions with a significant high-frequency peak of the spectrum in the low-TR data were also characterized with a flatter spectrum in the high-TR data. This can be explained with the undersampling of high-frequency fluctuations leading to spurious perceived oscillations at all frequency ranges – thus increasing the amplitude at all frequencies of the high-TR spectra more or less uniformely. In these regions, the true low-frequency fluctuations are therefore more difficult to discern amidst this nuisance variability, leading to increased functional contrast and thus differentiation of different brain networks with low-TR data.

A specific peak at higher frequencies, however, is not the only source of nuisance variability that was observed in our data. Some grey matter spectra, while having a clear low-frequency peak, converge to a higher baseline amplitude at higher frequencies than others. For example, the ratio between the maximum power at the low-frequency peak and the minimum power at higher frequencies (above 0.25 Hz) is 17.8 for the PCC, but 7.7 for the visual cortex, and is reduced even to 4.8 and 4.6 in the insula and the basal ganglia, respectively. In the amygdala, this ratio is only 3.2, lower than the ratio found in the ventricles, 4.2– only the white matter signal, with a ratio of 2.4, was below this level. This consistently higher power of fluctuations at frequencies that cannot be critically sampled with high-TR measurements leads to a constant increase of the amplitude of the spectrum across all frequencies, and thus a loss of functional contrast and specificity (see table 2). Therefore, when measuring with high TR EPI, in regions that show high baseline fluctuation amplitude at higher frequencies when measured with low TR, the true fluctuations at the frequencies actually sampled are hidden by this effect and cannot adequately be investigated.

One of the effects of this obfuscation can be seen in the low-frequency connectivity maps of the basal ganglia in figure 3. The basal ganglia are one of the regions with the lowest ratios between the low-frequency peak amplitude and the high-frequency baseline amplitude. While its low-frequency fluctuations in the low-TR dataset exhibit significant connectivity to other regions of the brain, including cerebellum and SMA, this information is lost in the high-TR dataset, where only connectivity within the (ipsi- and contralateral) basal ganglia is detected, analogously to previous publications on a basal ganglia resting-state network [22], [24].

Besides the problems of analyzing low-frequency fluctuations in the presence of undersampled high-frequency fluctuations, the high-frequency fluctuations themselves obviously also cannot be investigated with a high TR, as they cannot be critically sampled. Since low-TR scans have not yet been studied as thoroughly as high-TR scans, relatively little is known about the nature and the interpretation of high-frequency BOLD fluctuations. The presence of functional connectivity in the higher frequency bands hints at the possibility that these fast oscillations are not merely constituted of irrelevant noise. Indeed, when applying ICA to the high-passed time courses (above 0.25 Hz) of a subset of the low-TR dataset used here, consisting of the first 10 subjects measured, a number of large-scale components, including some of the typical resting-state networks – known for their characteristic low-frequency fluctuations – can be consistently identified [19].

An important point to note when interpreting these results is their heterogeneity across the brain. As seen in figure 1, there are some grey matter regions consistent with the assumption that fluctuations there can be seen as predominantly low-frequency effects, whereas others have a markedly higher proportion of high-frequency oscillations. The former group consists mainly of prefrontal and parietal cortices as well as the PCC, and to a lesser extent the occipital cortex and the remainder of the cingulate and frontal cortices. Grey matter regions with a larger amount of high-frequency fluctuations are subcortical regions as well as the insula, potentially due to contamination from adjacent vessels and CSF. On first glance, this would mean that the first of these groups of grey matter regions can adequately be studied with high-TR measurements, while the second cannot. However, it should be considered that connectivity between regions of the first and the second group will also be distorted in data originating from high-TR measurements, and thus cannot adequately be assessed either. This is a serious limitation of high-TR scans, in particular since subcortical regions play an important role in many neuroimaging studies and are known to connect to various cortical and cerebellar regions.

Even beyond the difficulties in investigating low frequency fluctuations in regions with a significant contribution of higher frequencies to the spectrum, the loss of high-frequency information itself can also hamper the exploration of regions with a more marked spectral low-frequency peak. In the connectivity analyses shown in figure 5, for almost all cortical regions there are significant connectivities that can be seen above 0.25 Hz, in the case of the motor cortex even above 0.75 Hz. Even though these higher frequency ranges contain less information than the lower frequencies, it seems prudent not to discard them too easily. At the low TR used here, it can be concluded that frequencies of at least up to 0.25 Hz contain low-frequency fluctuations specific to grey matter fluctuations and should therefore be considered in any analysis aimed at grey matter signals or networks.

Interestingly, these high-frequency connectivity results can be directly compared with the connectivities of white matter and CSF seeds. In the white matter, significant connectivity is only observed in the lowest frequency range (<0.1 Hz), with no connectivity at all at any higher frequency. In the CSF, there was no significant connectivity at any frequency. These results strongly suggest that, even though high-frequency oscillations are more prevalent in white matter and CSF, the patterns described here in terms of connectivity are highly specific to the grey matter. Even when comparing grey matter regions, there is the noteworthy tendency that the regions with the greatest magnitude of high-frequency fluctuations are not necessarily the regions where these fluctuations seem most structured (i.e., correlated to each other). On the contrary, regions with a low amplitude of high-frequency oscillations tend to have stronger connectivity patterns at these high frequencies. One interpretation of this effect might be that in regions with a higher proportion of power in the high frequency range of the spectrum, this power seems to be due to physiologic signals which mask underlying fluctuations of lower amplitude but possibly related to neuronal processes. Thus, in regions where such physiological high-frequency oscillations are absent, connectivity patterns can more readily be observed, even using high-TR protocols.

This study describes the differences in resting-state fluctuations at various frequencies in all areas of the brain across a wider frequency range than previous studies of up to 1.4 Hz. Two main aspects of high-frequency fluctuations can be distinguished. The first could best be described as high-frequency noise which overshadows the low-frequency fluctuations when undersampled, thus hampering the analysis of the affected regions with high-TR scans. The second are high-frequency fluctuations that are specific to the grey matter, which should be investigated in more detail in the future. Both of these aspects exhibit some variability across different brain regions, leading among others to the conclusion that there are also differences in how readily these regions can be studied using fMRI [23]. In terms of practical implications, this study demonstrates that low-TR fMRI scanning has considerable advantages over typical high-TR acquisition, since the latter not only miss out on relevant high-frequency fluctuations, but are also irreversibly perturbed by high-frequency fluctuations of non-neuronal origin obfuscating the actual signal of interest.

## Materials and Methods

### Low-TR Measurements

20 subjects (9 males/11 females, mean age 24.0, SD 2.9 years) recruited at Medical University of Vienna (MUV) gave written informed consent to participate in this fMRI study. The study protocol was approved by the Ethics Committee (EC) of the MUV according to the Declaration of Helsinki (2008). All participants were financially compensated for their expenditure of time. Prior to scanning, they underwent a comprehensive clinical assessment comprising previous history, neurological and medical examinations involving electrocardiography (ECG), blood pressure measurement and routine laboratory testing. Exclusion criteria were current or lifetime major medical and psychiatric disorders, clinically significant abnormalities in routine laboratory screening or general physical examination, previous serious head injury or loss of consciousness as well as the usual exclusion criteria for MR studies. Subjects underwent a 6 minute resting-state scan on a Siemens TIM Trio (3 T) scanner using a 32-channel head coil with a multiplexed EPI sequence [12], acquiring in total 1024 volumes (TE/TR = 32/354 ms, flip angle = 30°, 2.4×1.9×3.5 mm, bandwidth = 1748 Hz/pixel, 20 slices, 2 mm slice gap, multiband acceleration factor 4, 6/8 partial fourier). Subjects were instructed to keep their eyes closed, refrain from movement during the scan and avoid to fall asleep without concentrating on anything in particular. After the resting-state scan, a high-resolution anatomical image was acquired using MPRAGE with 1×1×1.1 mm resolution with 160 sagittal slices (TE/TR = 4.21/2300 ms, flip angle 9°, inversion time 900 ms).

### High-TR Measurements

20 additional subjects (10 males/10 females, mean age 25.3, SD 3.4 years) were recruited as described above and underwent standard, high-TR fMRI scans that were used as a reference for comparison with the low-TR measurements. Measurements were performed on the same Siemens TIM Trio scanner and 32-channel head coil. 167 volumes were acquired (TE/TR = 38/1800 ms, flip angle = 90°, 1.5×1.5×3 mm, bandwidth = 1446 Hz/pixel, 23 slices, 1.8 mm slice gap) using identical instructions to the subjects as for the low-TR measurements.

### Preprocessing

All data were preprocessed with a combination of AFNI [25] and FSL [26], using an analysis framework in R [27], [28] on Ubuntu Linux (Version 11.10 ‘Oneiric Ocelot’). Anatomical images were skull-stripped and normalized to MNI152 standard space. Functional images were corrected for intensity inhomogeneity using a bias field estimation by FSL FAST, skull-stripped and realigned to the 500th volume (of 1000) in order to minimize average displacement. Subsequently, functional images were aligned to the anatomical images in MNI152 standard space and resampled to 2×2×2 mm isotropic resolution, and motion parameters (3 translations and 3 rotations) were regressed out using a generalized linear model (GLM).

### Frequency Maps

At single-subject level, voxelwise discrete Fourier transformation was applied to obtain the power spectrum, and the proportion of spectral power in four frequency bands was computed. The lowest frequency band encompassed the frequencies up to 0.1 Hz, the range typically investigated in resting-state fMRI analysis, the second frequency band ranged from 0.1 to 0.25, which is the maximum frequency critically sampled with a typical TR of 2 s, the third and fourth frequency ranges comprised the high frequencies, with the threshold to separate them set at 0.75 Hz, which is about half the maximum frequency critically sampled with the low-TR protocol. The fraction of spectral power in all four frequency bands was computed for the low-TR data, and for the first two frequency bands for the high-TR data. These maps were then averaged between subjects for each dataset and each frequency band, resulting in 6 fractional amplitude maps. In addition, a fracional amplitude map of the two low-frequency bins combined (i.e., <0.1 Hz and 0.1–0.25 Hz) was calculated for comparison purposes. Finally, mean fractional amplitude values for all frequency bins were computed within grey matter, white matter and ventricle masks. To this end, individual subject masks were computed based on segmentation by FSL FAST, and in the case of white matter and ventricles, the intersection of this mask with prior masks obtained from the 1000 Functional Connectomes project were computed as the single-subject masks. For each subject, the mean fractional amplitudes of grey matter, white matter and ventricles were calculated for all frequency bins, and the resulting values then averaged across subjects.

### Regions of Interest

Nine regions of interest (ROIs) were selected for a more detailed presentation of power spectra, five of them in the cortical grey matter – posterior cingulate cortex (PCC) and medial prefrontal cortex (mPFC), both of them being parts of the default mode network (DMN), as well as the visual cortex, the motor cortex, and the insula – two in subcortical nuclei – one in the basal ganglia, one in the amygdala – and finally, one ROI in the white matter and one in the ventricles (see table 3 for coordinates). For each of these ROIs, the power spectrum of the center voxel was computed along with the ratio between the peak amplitude and the minimum amplitude to the right of the peak as a measure for the range of spectral amplitude. To allow for better comparisons, the spectra are normalized to the same area under the curve in the resulting figure.

**Table 3:** ROI locations.

ROI	x	y	z
PCC	0	62	32
mPFC	0	−58	12
Visual cortex	0	92	6
Motor Cortex	38	26	64
Insula	40	−4	−8
Basal ganglia	−24	−10	0
Amygdala	18	6	−22
Ventricle	0	2	18
White matter	−22	4	36

Location of ROI center voxels in MNI coordinates.

### Functional Connectivity

The same 9 ROIs were also used for a functional connectivity analysis. To this end, functional images were blurred with an isotropic Gaussian 6 mm FWHM kernel and bandpassed to each of the frequency bands used in the frequency maps (0–0.1 Hz, 0.1–0.25 Hz, 0.25–0.75 Hz, and 0.75 Hz−1.4 Hz for the low TR data, and only the first two of these bands for the high-TR data). Seed time series were extracted from each of these frequency bands, leading to 4 seed time courses per subject in the low-TR dataset and 2 seed time courses per subject in the high-TR dataset. Each of these seed time courses was the mean time series from a spherical region with a radius of 5 mm (containing 81 voxels) centered around the coordinates given in table 3. For both the high- and low-TR datasets and each of the single-band seeds, voxelwise functional connectivity was calculated for each subject, and these maps were subsequently averaged across the subjects of each of the two datasets to obtain group connectivity maps.

## References

[1] Biswal B, Yetkin FZ, Haughton VM, Hyde JS (1995) Functional connectivity in the motor cortex of resting human brain using echo-planar MRI. Magn Reson Med 34: 537–541. 8524021 10.1002/mrm.1910340409

[2] Biswal BB, Mennes M, Zuo XN, Gohel S, Kelly C, et al (2010) Toward discovery science of human brain function. Proc Natl Acad Sci U S A 107: 4734–4739. 20176931 10.1073/pnas.0911855107 PMC2842060

[3] Kalcher K, Huf W, Boubela RN, Filzmoser P, Pezawas L, et al (2012) Fully exploratory network independent component analysis of the 1000 functional connectomes database. Front Hum Neurosci 6: 301. 23133413 10.3389/fnhum.2012.00301 PMC3490136

[4] Kim SG, Ogawa S (2012) Biophysical and physiological origins of blood oxygenation leveldependent fMRI signals. J Cereb Blood Flow Metab 32: 1188–1206. 22395207 10.1038/jcbfm.2012.23 PMC3390806

[5] Aguirre GK, Zarahn E, D’esposito M (1998) The variability of human, BOLD hemodynamic responses. Neuroimage 8: 360–369. 9811554 10.1006/nimg.1998.0369

[6] Cunnington R, Windischberger C, Deecke L, Moser E (2002) The preparation and execution of self-initiated and externally-triggered movement: a study of event-related fMRI. Neuroimage 15: 373–385. 11798272 10.1006/nimg.2001.0976

[7] Cordes D, Haughton VM, Arfanakis K, Carew JD, Turski PA, et al (2001) Frequencies contributing to functional connectivity in the cerebral cortex in “resting-state” data. AJNR Am J Neuroradiol 22: 1326–1333. 11498421 PMC7975218

[8] Windischberger C, Cunnington R, Lamm C, Lanzenberger R, Langenberger H, et al (2008) Timeresolved analysis of fMRI signal changes using brain activation movies. J Neurosci Methods 169: 222–230. 18207248 10.1016/j.jneumeth.2007.11.033

[9] Sabatinelli D, Lang PJ, Bradley MM, Costa VD, Keil A (2009) The timing of emotional discrimination in human amygdala and ventral visual cortex. J Neurosci 29: 14864–14868. 19940182 10.1523/JNEUROSCI.3278-09.2009 PMC2821870

[10] Van Oort E, Norris D, Smith SM, Beckmann C (2012) Resting state networks are characterized by high frequency BOLD uctuations. In: OHBM. Abstract Nr. 739.

[11] Niazy RK, Xie J, Miller K, Beckmann CF, Smith SM (2011) Spectral characteristics of resting state networks. Prog Brain Res 193: 259–276. 21854968 10.1016/B978-0-444-53839-0.00017-X

[12] Feinberg DA, Moeller S, Smith SM, Auerbach E, Ramanna S, et al (2010) Multiplexed echo planar imaging for sub-second whole brain fMRI and fast diffusion imaging. PLoS One 5: e15710. 21187930 10.1371/journal.pone.0015710 PMC3004955

[13] Moeller S, Yacoub E, Olman CA, Auerbach E, Strupp J, et al (2010) Multiband multislice GE-EPI at 7 tesla, with 16-fold acceleration using partial parallel imaging with application to high spatial and temporal whole-brain fMRI. Magn Reson Med 63: 1144–1153. 20432285 10.1002/mrm.22361 PMC2906244

[14] Zahneisen B, Grotz T, Lee KJ, Ohlendorf S, Reisert M, et al (2011) Three-dimensional MRencephalography: fast volumetric brain imaging using rosette trajectories. Magn Reson Med 65: 1260–1268. 21294154 10.1002/mrm.22711

[15] Boyacioglu R, Barth M (2012) Generalized INverse imaging (GIN): Ultrafast fMRI with physiological noise correction. Magn Reson Med epub ahead of print. 10.1002/mrm.24528 23097342

[16] Smith SM, Miller KL, Moeller S, Xu J, Auerbach EJ, et al (2012) Temporally-independent functional modes of spontaneous brain activity. Proc Natl Acad Sci U S A 109: 3131–3136. 22323591 10.1073/pnas.1121329109 PMC3286957

[17] Zuo XN, Xu T, Jiang L, Yang Z, Cao XY, et al (2013) Toward reliable characterization of functional homogeneity in the human brain: preprocessing, scan duration, imaging resolution and computational space. Neuroimage 65: 374–386. 23085497 10.1016/j.neuroimage.2012.10.017 PMC3609711

[18] Liao XH, Xia MR, Xu T, Dai ZJ, Cao XY, et al (2013) Functional brain hubs and their test-retest reliability: a multiband resting-state functional MRI study. Neuroimage 83: 969–982. 23899725 10.1016/j.neuroimage.2013.07.058

[19] Boubela RN, Kalcher K, Huf W, Kronnerwetter C, Filzmoser P, et al (2013) Beyond noise: Using temporal ICA to extract meaningful information from high-frequency fMRI signal uctuations during rest. Front Hum Neurosci 7: 168. 23641208 10.3389/fnhum.2013.00168 PMC3640215

[20] Baria AT, Baliki MN, Parrish T, Apkarian AV (2011) Anatomical and functional assemblies of brain BOLD oscillations. J Neurosci 31: 7910–7919. 21613505 10.1523/JNEUROSCI.1296-11.2011 PMC3114444

[21] He BJ, Zempel JM, Snyder AZ, Raichle ME (2010) The temporal structures and functional significance of scale-free brain activity. Neuron 66: 353–369. 20471349 10.1016/j.neuron.2010.04.020 PMC2878725

[22] Robinson S, Basso G, Soldati N, Sailer U, Jovicich J, et al (2009) A resting state network in themotor control circuit of the basal ganglia. BMC Neurosci 10: 137. 19930640 10.1186/1471-2202-10-137 PMC2785820

[23] Boubela RN, Kalcher K, Nasel C, Moser E (2014) Scanning fast and slow: current limitations of 3 tesla functional MRI and future potential. Front Physics 2: 1. 10.3389/fphy.2014.00001 PMC5291320 28164083

[24] Schöpf V, Kasess CH, Lanzenberger R, Fischmeister F, Windischberger C, et al (2010) Fully exploratory network ICA (FENICA) on resting-state fMRI data. J Neurosci Methods 192: 207–213. 20688104 10.1016/j.jneumeth.2010.07.028

[25] Cox RW (1996) AFNI: software for analysis and visualization of functional magnetic resonance neuroimages. Comput Biomed Res 29: 162–173. 8812068 10.1006/cbmr.1996.0014

[26] Smith SM, Jenkinson M, Woolrich MW, Beckmann CF, Behrens TEJ, et al (2004) Advances in functional and structural MR image analysis and implementation as FSL. Neuroimage 23 Suppl 1S208–S219. 15501092 10.1016/j.neuroimage.2004.07.051

[27] R Development Core Team (2013) R: A language and environment for statistical computing.

[28] Boubela RN, Huf W, Kalcher K, Sladky R, Filzmoser P, et al (2012) A highly parallelized framework for computationally intensive MR data analysis. Magn Reson Mater Phy 25: 313–320. 10.1007/s10334-011-0290-7 22086306

